# Mortality Prediction in Diffuse Large B-Cell Lymphoma Using Supervised Machine Learning Models—A Retrospective Study

**DOI:** 10.3390/jcm14228216

**Published:** 2025-11-19

**Authors:** Cosmin-Daniel Minciuna, Dorina Minciuna, Angela-Smaranda Dascalescu, Amalia Titieanu, Vlad-Andrei Cianga, Ion Antohe, Ingrid-Andrada Vasilache, Catalin-Doru Danaila, Lucian Miron

**Affiliations:** 1 Department of Hematology, Grigore T. Popa University of Medicine and Pharmacy, 700115 Iasi, Romania; 2 Department of Mother and Child Care, Grigore T. Popa University of Medicine and Pharmacy, 700115 Iasi, Romania; 3 Department of Medical Oncology-Radiation Therapy, Faculty of Medicine, Grigore T. Popa University of Medicine and Pharmacy, 700115 Iasi, Romania

**Keywords:** diffuse large B-Cell lymphoma, prognosis, machine learning, prediction, death

## Abstract

**Background/Objectives**: Diffuse large B-cell lymphoma (DLBCL) is a biologically and clinically heterogeneous malignancy with variable outcomes. Accurate risk prediction at diagnosis remains essential to guide treatment and follow-up strategies. In this retrospective study we aimed to assess the performance of multiple modeling approaches to predict death by 26 months of follow-up in patients with DLBCL using data available in the diagnostic stage. **Methods**: In this study we included 412 patients with DLBCL who were evaluated, treated, and followed-up at the Regional Institute of Oncology in Iasi, Romania, between 2015 and 2023. Clinical and paraclinical data determined at baseline examination was used to train and test six machine learning models (logistic regression, random forest—RF, support vector machine with a radial-basis kernel—SVM-RBF, multilayer perceptron neural network—MLP, random survival forest—RSF, and extreme gradient boosting—XGBoost) and to compare their performance to the Cox proportional hazards model. **Results**: Among the models, RF achieved the highest discrimination (AUC = 0.9060), with balanced performance (accuracy = 0.833; F1 = 0.902), followed by XGBoost (AUC = 0.8335) and MLP (AUC = 0.7861; accuracy = 0.849). RF and logistic regression demonstrated the best calibration (Brier = 0.360 and 0.377). The Cox model achieved moderate discrimination (time-dependent AUC = 0.5561; C-index = 0.55). **Conclusions**: Our findings align with contemporary reports showing that machine learning frameworks can outperform classical prediction approaches.

## 1. Introduction

Diffuse large B-cell lymphoma (DLBCL) is a highly heterogeneous and aggressive type of non-Hodgkin lymphoma (NHL) with variable survival outcomes. The projected 10-year prevalence of DLBCL was reported to increase in the United States and Western Europe from 2020 to 2025 [1]. In the United States, prevalence was projected to rise from 162,604 in 2020 to 182,433 in 2025, while in Western Europe, it was projected to rise from 143,286 to 153,930. Also, the projected incidence was likewise expected to grow over the same period. In the United States, incident cases were projected to increase from 29,108 in 2020 to 32,443 in 2025. In Western Europe, incidence was projected to increase from 26,078 to 27,981 [1].

Recent statistics from the National Cancer Institute within the Surveillance, Epidemiology, and End Result Program (SEER) showed a rate of new cases of 5.6 per 100,000 men and women per year, as well as a death rate of 1.7 per 100,000 men and women per year, using data from 2018 to 2022 [2].

Accurate prediction of death risk is essential for guiding treatment and for improving prognosis. Among risk factors that can influence the prognosis are disease stage and bulky mass, International Prognostic Index (IPI) and its variants, which include age, ECOG (Eastern Cooperative Oncology Group) status, stage, serum lactate dehydrogenase (LDH), and extranodal sites; double/triple-hit status (translocations involving *MYC*, *BCL2*, and/or *BCL6*); genetic mutations (*CREBBP*, *EP300*, *MYD88*, *CD79B*, *SOCS1*); presence of B symptoms (unexplained fever > 38 °C, drenching night sweats, and unintentional weight loss > 10% over 6 months); cell-of-origin (ABC, activated B-cell-like); double-expressor/double-hit biology; high expression of Ki67; bone marrow or CNS involvement; and early treatment response [3,4,5,6,7,8,9,10,11,12,13].

Recent research based on artificial intelligence (AI) has advanced beyond traditional clinical indices, integrating molecular, genetic, imaging, and liquid biopsy markers to enhance death prediction in DLBCL. These tools offer improved accuracy and individualized risk stratification compared to traditional methods, supporting individualized care for DLBCL patients. The AI-based models used to predict survival in DLBCL patients include ensemble learning (stacking, extreme gradient boosting—XGBoost, random forest-RF, etc.), neural networks, or autoencoders and transformer-based models [14,15,16,17,18,19,20].

Although the majority of data from the literature refers to the use of AI-based models for determining the biomolecular and genetic signature of DLBCL [21,22,23,24,25], very few concentrate on the prediction of specific disease outcomes using clinical and paraclinical data in this category of patients. Ismayilov et al. [26] conducted a retrospective cohort study for the prediction of CNS relapse in DLBCL patients using random survival forests (RSFs) and gradient boosting machines (GBMs). Their results indicated that RSFs achieved the highest discriminative power (C-index: 0.91), outperforming traditional scores such as IPI scores. Extranodal site number, high-risk organ involvement, and ECOG performance status were the predictors with the highest influence on the model’s performance [26]. Also, a multicenter retrospective analysis of 836 DLBCL patients under 18 years old tested the performance of a machine learning model that combined XGBoost with Cox and generalized Cox regression models for the prediction of disease prognosis, achieving AUC values of above 0.7 [27].

The primary objective of this retrospective study was to assess the performance of multiple modeling approaches to predict death by 26 months of follow-up in patients with DLBCL, using data available in the diagnostic stage.

## 2. Materials and Methods

We conducted a retrospective cohort study using clinical database of 412 patients with DLBCL who were evaluated, treated, and followed-up at the Regional Institute of Oncology in Iasi, Romania, between 2015 and 2023. Ethical approval for this study was obtained from the Institutional Ethics Committee of the hospital (No. 197/3 July 2023) and of Grigore T. Popa University of Medicine and Pharmacy, Iasi, Romania (No. 484/28 October 2024).

The inclusion criteria were a pathologically confirmed diagnosis of DLBCL; complete, routinely collected clinical, laboratory, and imaging data; and informed consent for medical data processing. We excluded patients with non-DLBCL histology, primary CNS lymphoma, and incomplete medical records or no determinable 26-month status.

Medical records of the included patients were evaluated and the following data was recorded for the purpose of this study: demographics (age, sex), comorbidity burden, prior neoplasia, viral hepatitis and HIV status, disease characteristics (stage, B symptoms, extranodal involvement, histologic transformation, IPI, CNS-IPI, ECOG performance status, LDH, Ki67, cell-of-origin subtype), and treatment/response variables (chemotherapy exposure, number of cycles, treatment completion, interim and end-of-treatment CT (computed tomography) and PET-CT (positron emission tomography–computed tomography) assessments, first-line response, primary refractory status, radiotherapy).

All models were trained using baseline demographic, clinical, and biological predictors available at the time of diagnosis, including sex, age, comorbidities, stage, ECOG performance status, LDH, IPI, CNS-IPI class, extranodal involvement, and molecular subtype (GCB/ABC, double-expressor status, Ki67 index).

Post-treatment assessments (e.g., interim or final imaging response, chemotherapy response, refractory status) were excluded from analysis to prevent information leakage and ensure that predictions reflected pre-treatment decision-making.

For binary prediction tasks, the endpoint was death by 26 months after diagnosis (primary outcome). Missing values in modeling were addressed during preprocessing and standardization by median imputation for continuous predictors and mode imputation for categorical predictors. A table with the missing data is presented in the Supplementary Material (Table S1. Missing Values per Column). Continuous variables were standardized (mean = 0, SD = 1), and categorical variables were one-hot encoded, accounting for possible unseen levels in the test set.

The dataset was split into a training (80%) and a held-out test set (20%), using stratified random sampling based on the outcome variable. All preprocessing steps were fit on the derivation set only and then applied to the held-out cohort to prevent information leakage.

Five supervised machine learning algorithms were trained to predict the outcome: logistic regression, random forest (RF), support vector machine with radial basis function kernel (SVM-RBF), Multilayer Perceptron (MLP) neural network, and extreme gradient boosting (XGBoost).

All models used the same set of baseline predictors and were trained to optimize binary cross-entropy loss. Class imbalance was addressed using class-weight adjustment within each model. Hyperparameters were optimized via grid search and validated by stratified five-fold cross-validation.

Performance was assessed on the held-out test cohort using accuracy, area under the receiver operating characteristic curve (AUC), sensitivity, specificity, and F1-score. Feature importance for the best performing model was also assessed.

Model calibration was evaluated using Brier scores and calibration curves. Clinical utility was assessed through decision curve analysis (DCA) by estimating the net benefit across a range of probability thresholds. Calibration curves were generated using both isotonic regression and decile grouping for visual assessment.

To complement the classification analysis, two time-to-event models were constructed using the same baseline variables: a Cox proportional hazards (Cox PH) model and an RSF model. The Cox PH model was fitted using the partial likelihood method with proportional hazards assumption verified via Schoenfeld residuals. The RSF model employed 1000 trees with log-rank splitting and out-of-bag estimation for internal validation. Model performance was quantified using the time-dependent AUC, Harrell’s concordance index (C-index), and Integrated Brier Score (IBS) across 20–100 months.

Kaplan–Meier curves were generated for outlining overall survival with numbers at risk. Cumulative incidence of death (1−S(t)) was tabulated at prespecified times. Continuous variables were summarized as means (±standard deviation, SD) and compared with *t*-tests or Wilcoxon tests as appropriate. Categorical variables were compared with chi-square or Fisher’s exact tests. Two-sided *p*-values < 0.05 were considered statistically significant.

Analyses were performed in Python version 3.12 (Python Software Foundation, Beaverton, OR, USA) and Stata version 19.5 (StataCorp, College Station, TX, USA) for Kaplan–Meier summaries.

## 3. Results

### 3.1. Baseline Characteristics of the Study Population

Patients who died were significantly older at diagnosis compared with survivors (mean 65.6 vs. 58.5 years, *p* < 0.001). Sex distribution was similar between groups, with no significant association with survival. Comorbidities were more frequent among deceased patients (80.2% vs. 70.2%, *p* = 0.020). The presence of previous neoplasia, viral hepatitis (HBV or HCV), and HIV infection showed no significant differences between groups.

Stage distribution did not differ significantly, although a higher proportion of survivors were diagnosed at stages I–II. The presence of B symptoms was more common among deceased patients (65.5% vs. 54.4%, *p* = 0.022). The IPI distribution was significantly worse in the deceased group (*p* < 0.001). Extranodal involvement and histologic transformation did not differ significantly. However, bulky disease was more frequent among survivors (68.4% vs. 58.9%, *p* = 0.045).

ECOG performance status differed markedly: deceased patients had poorer scores, with 28.5% having an ECOG status of 3–4 compared to only 7.4% of survivors (*p* < 0.001). LDH levels were also strongly associated with mortality: patients with elevated LDH were overrepresented among the deceased (*p* < 0.001). Bone marrow evaluation and infiltration did not reach statistical significance. CNS-IPI risk class was strongly associated with outcome, with survivors more frequently classified as low or intermediate risk (*p* < 0.001).

Gastric involvement did not differ significantly between groups. GCB and ABC subtypes showed borderline differences (*p* = 0.052), with GCB more frequent among survivors. Double expresser status was not significantly associated with survival. Ki67 proliferation index was slightly higher in survivors (44.5 vs. 36.9, *p* = 0.029).

Interim CT assessments showed clear prognostic value (*p* < 0.001): survivors more often achieved partial or complete responses, while progression was more frequent among the deceased. Final CT and PET evaluations confirmed these findings, with complete response strongly associated with survival (*p* < 0.001).

First-line chemotherapy response was also strongly predictive: 87.4% of survivors achieved complete response compared with only 22.3% of deceased patients (*p* < 0.001). Finally, primary refractory disease was strongly linked to mortality: 91.2% of survivors were non-refractory, compared with only 32.5% among the deceased (*p* < 0.001) (Table 1).

### 3.2. Kaplan–Meier Analysis

**Figure 1:**
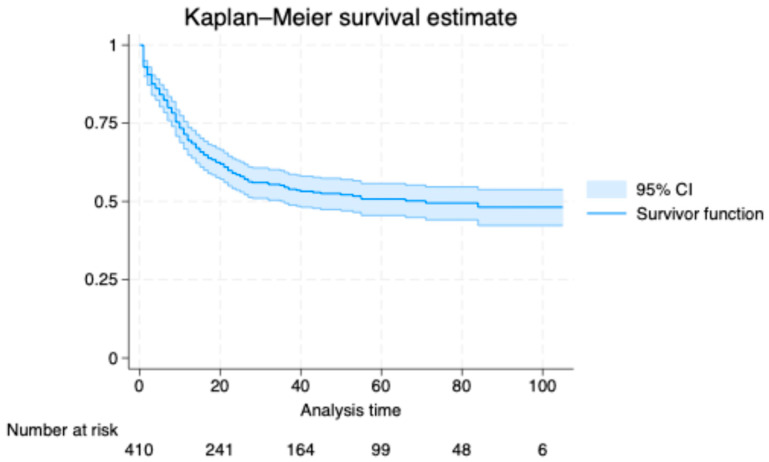
Kaplan–Meier survival estimates.

In the Kaplan–Meier analysis, the estimated cumulative incidence of death was 38.0% at 20 months (95% confidence interval, CI, 33.5 to 42.9), 46.8% at 40 months (95% CI, 41.9 to 51.9), 49.3% at 60 months (95% CI, 44.3 to 54.5), 50.5% at 80 months (95% CI, 45.4 to 55.9), and 51.8% at 100 months (95% CI, 46.2 to 57.7). The survival curve declined steeply early and then flattened, indicating a higher early hazard that attenuated with time (Figure 1). The median survival time was 20 months. Numbers at risk decreased from 410 at baseline to 241, 164, 99, 48, and 6 at 20, 40, 60, 80, and 100 months, respectively.

### 3.3. Performance Metrics of Classification Models

The performance metrics of the classification models for death prediction in our cohort of patients using baseline predictors is presented in Table 2. Our data indicated that across all classification models, accuracy ranged from 0.823 to 0.849, AUC values from 0.741 to 0.906, and F1-scores from 0.896 to 0.909.

The RF classifier achieved the highest discrimination (AUC = 0.9060) and maintained balanced predictive performance (accuracy = 0.833; F1 = 0.902). Its sensitivity was high (0.974), but its specificity reached 0.30 for identifying patients who died within 26 months.

The XGBoost model performed comparably (AUC = 0.8335; accuracy = 0.839; F1 = 0.903), with a sensitivity of 0.947 and a specificity of 0.42. Compared with RF, XGBoost slightly improved the rate of correctly identified survivors but at a marginal cost in sensitivity.

The MLP neural network achieved the highest accuracy overall (0.849) and the highest F1-score (0.909), with a sensitivity of 0.954 and a specificity of 0.45, indicating improved discrimination between outcome classes and a more balanced false-positive to false-negative ratio. However, its AUC of 0.7861 was lower than previous models, suggesting a more variable performance across probability thresholds.

**Figure 2:**
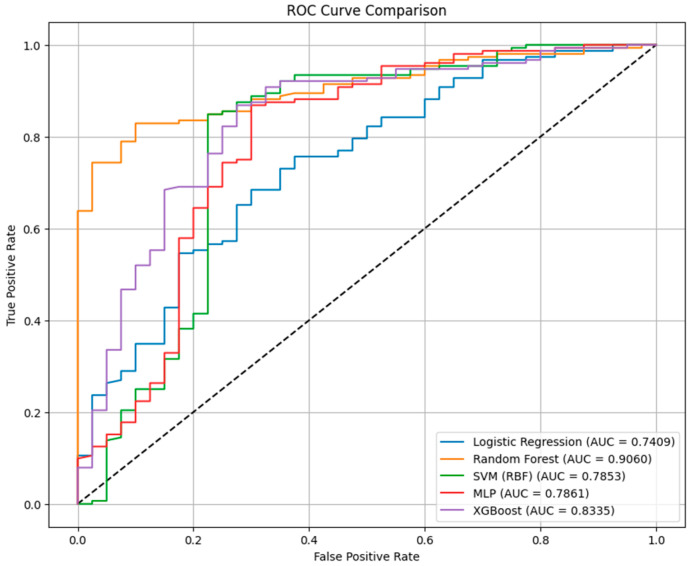
ROC curves comparison between the classification models at 26 months.

The SVM-RBF model produced an AUC of 0.7853, an accuracy of 0.839, and an F1 of 0.908, but was characterized by a high false-positive rate. On the other hand, the logistic regression model demonstrated the lowest AUC (0.7409) and specificity (0.28), but preserved high sensitivity (0.967) and an F1-score of 0.896, with an overall accuracy of 0.823. Figure 2 represents the Receiver Operating Characteristic (ROC) curve comparison between the classification models, considering death at 26 months as outcome.

**Figure 3:**
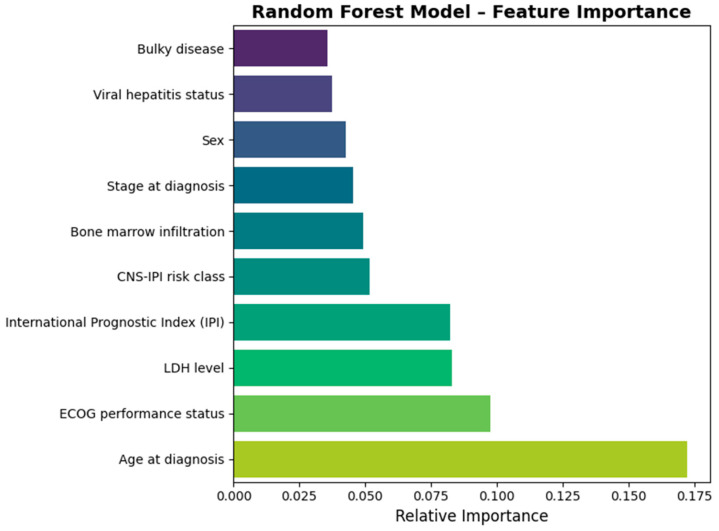
Feature importance for the best performing model.

Figure 3 outlines the most important predictors that in the best performing classification model, RF. Our data indicated that age at diagnosis, the ECOG performance status, LDH serum levels, IPI, and CNS-IPI risk class had the highest importance in the classification model.

### 3.4. Calibration Performance and Decision Curve Analysis

**Figure 4:**
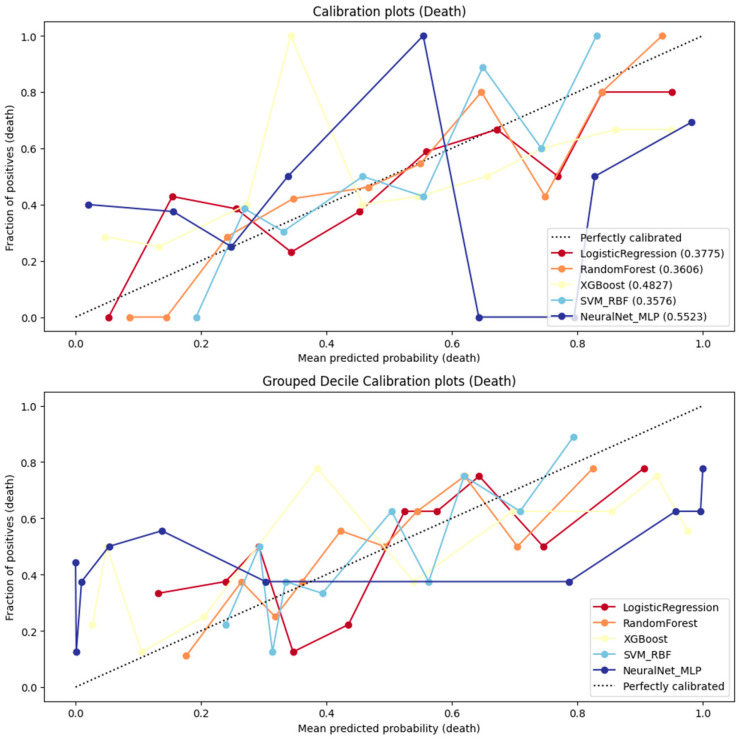
Calibration plots for all classification models (**top**: detailed; **bottom**: decile grouping).

Calibration assessment (Figure 4) demonstrated variable agreement between predicted and observed 26-month mortality probabilities across the evaluated models. Our data indicated that RF and logistic regression models exhibited the lowest Brier scores (0.360 and 0.377, respectively), indicating more accurate probability estimation.

The SVM-RBF model achieved a comparable Brier score (0.357), but showed greater fluctuation across risk intervals. XGBoost demonstrated moderate calibration (Brier = 0.482), with a tendency to overpredict mortality in intermediate-risk patients. In contrast, the MLP neural network displayed the highest prediction error (Brier score = 0.552) and inconsistent calibration, reflected by marked deviations from the reference line in both detailed and decile-grouped calibration curves.

**Figure 5:**
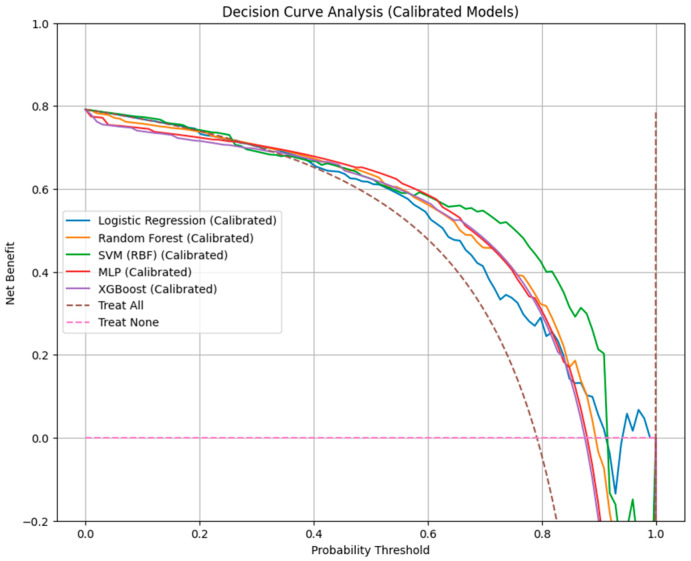
Decision curve analysis comparing net benefit across classification models.

Decision-curve analysis (Table 3 and Figure 5) further contextualized these findings in terms of clinical net benefit. All machine learning classifiers outperformed the “treat-all” and “treat-none” strategies across relevant threshold probabilities. The DCA threshold ranged between 0.10 and 0.60.

RF provided the broadest range of clinical utility (net benefit = 0.10–0.60), followed by logistic regression and SVM-RBF (0.10–0.50), suggesting that these models yield the most meaningful trade-off between benefit (true-positive detection) and harm (false-positive classification) in clinical decision-making.

While MLP and XGBoost achieved reasonable net benefit within similar ranges, their poorer calibration indicated reduced reliability of absolute risk estimation despite acceptable discrimination.

### 3.5. Survival Modeling Performance and Calibration

Table 4 presents the performance of Cox PH and RSF models that were developed using the same baseline predictors. When evaluated at the 26-month horizon, the Cox PH model demonstrated higher discrimination (time-dependent AUC = 0.5561, C-index = 0.548) compared with the RSF (AUC = 0.4099, C-index = 0.431). Calibration was similar, with nearly identical IBSs of 0.24 for both models and consistent pointwise Brier scores at 20, 40, and 60 months (0.25–0.27).

**Figure 6:**
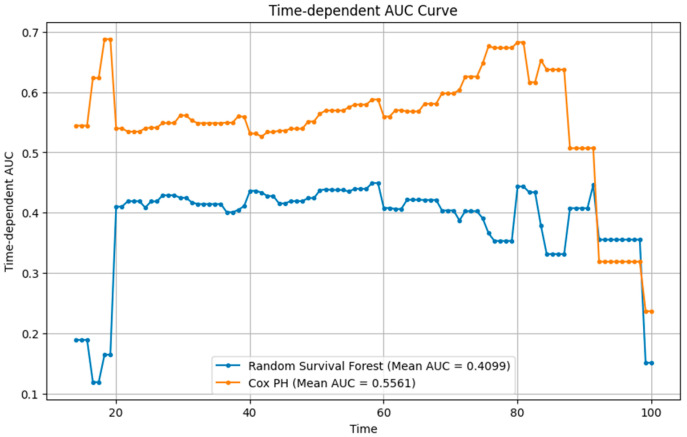
Time-dependent AUC (Cox PH vs. RSF).

The time-dependent AUC (Figure 6) showed that Cox PH maintained moderate but stable discrimination over time, while RSF exhibited early instability and overall lower performance.

## 4. Discussion

In this single-center retrospective cohort of 412 patients with DLBCL, we compared classical statistical and machine learning approaches for predicting death. Our findings showed that the RF model achieved the highest discrimination (AUC = 0.9060) and balanced performance and precision metrics (F1 = 0.902). Its high sensitivity (0.974) ensured that most deaths were correctly identified, although specificity remained modest (0.30), reflecting a trade-off between minimizing false negatives and accepting a higher false-positive rate. The MLP network achieved the highest accuracy (0.849) and F1-score (0.909), but its calibration was less reliable (Brier = 0.552), limiting its clinical interpretability. XGBoost demonstrated a comparable F1-score (0.903) and moderate calibration (Brier = 0.482). Finally, the logistic regression model, despite lower discrimination (AUC = 0.7409), maintained competitive sensitivity (0.967) and good calibration, confirming its ongoing relevance as a transparent, interpretable baseline model.

Calibration analyses demonstrated that RF and logistic regression provided the highest concordance between predicted and observed mortality probabilities, with the lowest Brier scores (0.360 and 0.377, respectively). Decision-curve analysis further indicated that RF achieved the broadest net-benefit range (0.10–0.60). Survival models demonstrated limited discrimination relative to classification models. The Cox PH model achieved modest but stable predictive accuracy (time-dependent AUC = 0.556, C-index = 0.55), outperforming the RSF (AUC = 0.409, C-index = 0.43). Integrated Brier scores were similar (0.24), indicating comparable calibration.

Feature importance analysis within the RF model confirmed that classical prognostic factors dominated prediction. The main contributors included age at diagnosis, ECOG performance status, LDH level, IPI score, and CNS-IPI class, which collectively accounted for more than half of model variance. Bone-marrow involvement, disease stage, sex, and viral hepatitis status had a modest effect on model performance.

Our data is similar to that published in recent papers which outlines the utility of complex machine learning models for risk stratifications as well as prognostic factors for DLBCL patients. Peng et al. reported that an AI-enabled prognostic framework integrating clinical and molecular features outperformed IPI for patients with DLBCL in a large, single-center Chinese cohort [14]. The authors used random survival forests to weight 22 candidate variables, and their “molecular-contained prognostic model” (McPM) achieved a higher C-index and lower integrated Brier score than IPI for both overall and progression-free survival, with superior ROC performance as well. Also, a simplified model (sMcPM) outlined five prognostic factors (number of extranodal sites, LDH, MYC status, absolute monocyte count, and platelet count) that achieved strong discrimination and stratified overall survival at least as well as IPI and markedly improved PFS stratification [14].

In a monocentric retrospective cohort of 130 adults, Detrait et al. evaluated five supervised algorithms to predict primary refractory DLBCL using routine clinical features, first-line therapy, and interim PET after two cycles [28]. Among 124 treated patients, the 3-year OS was 58.5%. Upon univariable analysis, age, Ann Arbor stage, comorbidity, IPI, first-line regimen, CMV/EBV infection, and socioeconomic status were associated with refractoriness. The Naïve Bayes Categorical classifier performed best (ROC-AUC 0.81, 95% CI: 0.64–0.96, accuracy 83%, F1 0.82, false-positive rate 10%), outperforming XGBoost (AUC: 0.74) and RF (AUC: 0.67) [28]. However, the limited number of patients included and the single-center design limit the generalizability of the results.

Moreover, Liu et al. [29] conducted a retrospective cohort study and developed a novel FLAMB prognostic model (ferritin, LDH, age, monocyte count, β2-microglobulin), which was constructed by integrating the Cox regression model with the random forest algorithm in order to predict survival in patients diagnosed with primary gastric DLBCL. Their results showed that the FLAMB model demonstrated superior discriminative power (C-index: 0.653 vs. 0.637, Δ = 1.6%) and more efficient classification of high-risk patients compared to the IPI score. This enhanced risk stratification was confirmed by a statistically significant log-rank test (*p* < 0.05). The authors outlined the clinical utility of this model for more precise prognostic stratification than the IPI, particularly in primary care or community hospitals [29].

Moreover, Fan et al. developed a staking predictive model to predict the mortality hazard of 406 DLBCL patients within 2 years of treatment using predictor variables selected by the Cox model, the logistic model, and the random forest algorithm [30]. Their results indicated that gender, stage, IPI, Karnofsky performance status, and rituximab were significant factors for predicting the deaths of DLBCL patients. The model achieved an AUC value of 0.820.

Krajnc et al. showed that clinician-driven data preprocessing can measurably improve oncology ML performance and introduced a rule set table and preference-based actions that were used for features or samples preprocessing steps [31]. Using XGBoost across prostate, glioma, and DLBCL cohorts, the authors demonstrated that the RST-guided preprocessing produced the largest gains in balanced accuracy, up to +18% in glioma, +6% in prostate, and +3% in DLBCL, compared with models without RST [31]. These findings support a clinician-based approach to preprocessing that can enhance the generalization of the findings.

Also, Luo et al. demonstrated that multi-view learning that uses PET-CT radiomics and clinical covariates can enhance prognostic modeling in DLBCL [32]. Their results showed that an SVM model using the integrated feature set performed best (AUC: 86.3%, accuracy: 91.6%, sensitivity: 83.2%, F1: 85.7%, G-mean: 86.1%) [32]. These findings support the premise that aligning clinical and imaging representations in a shared kernel subspace can capture complementary biology and yield superior discrimination.

The results from our study should be interpreted considering a series of limitations. They include the retrospective single-center design and potential selection and information biases. Moreover, our findings reflect internal validation only, and external validation across multiple centers and health systems is required to establish the generalizability and explicability of the models.

Future research should concentrate on improving the models’ discriminative power and should include molecular markers that can shift the feature importance from post-treatment assessments to diagnostic features. Considering this perspective, we support the inclusion of liquid biopsy markers and imaging parameters at diagnosis that can improve the overall performance of these models for the prediction of DLBCL prognosis.

## 5. Conclusions

In this single-center retrospective cohort study, we found that the RF model achieved the best overall balance between sensitivity and calibration, outperforming both classical statistical and other machine learning models.

Conventional predictors such as age, ECOG performance status, LDH, IPI, and CNS-IPI remained the dominant contributors to model performance, confirming the relevance of established prognostic indices, even in the context of advanced analytical frameworks.

While the Cox proportional hazards model exhibited stable time-dependent discrimination, its overall accuracy was modest compared with classification-based approaches, suggesting that machine learning models may better capture nonlinear interactions among prognostic features.

Logistic regression retained strong calibration and interpretability, supporting its utility as a clinically interpretable baseline model.

Future work should prioritize shifting predictive power toward pre-treatment features by incorporating baseline molecular surrogates and standardized imaging descriptors.

## Figures and Tables

**Table 1:** Comparison of general characteristics of the study groups.

Variable	Category	Deceased (n = 197)	Alive (n = 215)	*p*-Value
Age at diagnosis (years)	Mean ± SD	65.6 ± 14.2	58.5 ± 14.3	<0.001
Sex	Male	96 (48.7%)	96 (44.7%)	0.407
	Female	101 (51.3%)	119 (55.3%)	
Comorbidities	Yes	158 (80.2%)	151 (70.2%)	0.020
Previous neoplasia	Yes	12 (6.1%)	14 (6.5%)	0.861
Viral hepatitis	None	151 (76.7%)	174 (80.9%)	0.089
	HBV	27 (13.7%)	32 (14.9%)	
	HCV	19 (9.6%)	9 (4.2%)	
HIV	Yes	3 (1.5%)	2 (0.9%)	0.583
Stage at diagnosis	I	7 (3.6%)	14 (6.5%)	0.270
	II	30 (15.2%)	43 (20.0%)	
	III	31 (15.7%)	32 (14.9%)	
	IV	129 (65.5%)	126 (58.6%)	
B symptoms	Absent (A)	68 (34.5%)	98 (45.6%)	0.022
	Present (B)	129 (65.5%)	117 (54.4%)	
IPI (0–5 score)	Higher score = worse prognosis	significantly different distribution		<0.001
Extranodal involvement	Yes	121 (61.4%)	135 (62.8%)	0.775
Histologic transformation	Yes	6 (3.0%)	8 (3.7%)	0.706
Bulky disease	Yes	116 (58.9%)	147 (68.4%)	0.045
ECOG	0	10 (5.1%)	32 (14.9%)	<0.001
	1	71 (36.0%)	123 (57.2%)	
	2	60 (30.5%)	44 (20.5%)	
	3	36 (18.3%)	14 (6.5%)	
	4	20 (10.2%)	2 (0.9%)	
LDH	Normal	68 (34.5%)	131 (60.9%)	<0.001
	>ULN < 2 × ULN	59 (30.0%)	51 (23.7%)	
	2 × ULN	26 (13.2%)	12 (5.6%)	
	3 × ULN	6 (3.0%)	4 (1.9%)	
	>3 × ULN	38 (19.3%)	17 (7.9%)	
Bone marrow infiltration	Yes	35 (17.8%)	58 (27.0%)	0.082
CNS-IPI risk class	Low	16 (8.1%)	51 (23.7%)	<0.001
	Intermediate	96 (48.7%)	131 (60.9%)	
	High	85 (43.1%)	33 (15.3%)	
Gastric involvement	Yes	28 (14.2%)	41 (19.1%)	0.188
GCB subtype	Yes	33 (16.8%)	57 (26.5%)	0.052
	No	100 (50.8%)	100 (46.5%)	
	Missing	64 (32.5%)	58 (27.0%)	
ABC subtype	Yes	100 (50.8%)	100 (46.5%)	0.052
	No	33 (16.8%)	57 (26.5%)	
	Missing	64 (32.5%)	58 (27.0%)	
Double expresser	No	182 (92.4%)	195 (90.7%)	0.726
	Yes	13 (6.6%)	16 (7.4%)	
	Missing	2 (1.0%)	4 (1.9%)	
Ki67	Mean ± SD	36.88 ± 40.89	44.53 ± 40.68	0.029
Interim CT	Stable disease	1 (0.5%)	1 (0.5%)	<0.001
	Partial response	67 (34.0%)	121 (56.3%)	
	Complete response	11 (5.6%)	37 (17.2%)	
	Progressive disease	20 (10.2%)	4 (1.9%)	
	Not evaluated	98 (49.7%)	52 (24.2%)	
Final CT	Stable disease	1 (0.5%)	0 (0.0%)	<0.001
	Partial response	18 (9.1%)	14 (6.5%)	
	Complete response	42 (21.3%)	177 (82.3%)	
	Progressive disease	25 (12.7%)	5 (2.3%)	
	Not evaluated	102 (51.8%)	18 (8.4%)	
Final PET-CT	Stable disease	8 (4.1%)	13 (6.0%)	<0.001
	Partial response	17 (8.6%)	131 (60.9%)	
	Complete response	16 (8.1%)	10 (4.7%)	
	Progressive disease	155 (78.7%)	61 (28.4%)	
First-line CHT response	Stable disease	1 (0.5%)	0 (0.0%)	<0.001
	Partial response	18 (9.1%)	7 (3.3%)	
	Complete response	44 (22.3%)	188 (87.4%)	
	Progressive disease	80 (40.6%)	17 (7.9%)	
	Not evaluated	46 (23.4%)	3 (1.4%)	
	Not treated	8 (4.1%)	0 (0.0%)	
Primary refractory	No	64 (32.5%)	196 (91.2%)	<0.001
	Yes	95 (48.2%)	17 (7.9%)	
	Did not complete treatment	23 (11.7%)	1 (0.5%)	
	Not treated	11 (5.6%)	1 (0.5%)	
	Could not be evaluated	4 (2.0%)	0 (0.0%)	

Legend: HBV, hepatitis B virus; HCV, hepatitis C virus; HIV, human immunodeficiency virus; IPI, International Prognostic Index; ECOG, Eastern Cooperative Oncology Group performance status; LDH, lactate dehydrogenase; CNS-IPI, Central Nervous System International Prognostic Index; GCB, germinal center B-cell-like; ABC, activated B-cell-like; CT, computed tomography; PET-CT, positron emission tomography–computed tomography; CHT, chemotherapy; SD, standard deviation.

**Table 2:** Classification models’ performance for death prediction at 26 months in the evaluated cohort.

Model	Accuracy	AUC Value	Se	Sp	F1-Score
Logistic regression	0.823	0.7409	0.967	0.28	0.896
Random forest	0.833	0.9060	0.974	0.30	0.902
SVM-RBF	0.839	0.7853	1.000	0.23	0.908
MLP (Neural Net)	0.849	0.7861	0.954	0.45	0.909
XGBoost	0.839	0.8335	0.947	0.42	0.903

Legend: AUC, Area Under the ROC Curve; Se, sensitivity; Sp, specificity; XGBoost, extreme gradient boosting; SVM-RBF, support vector machine with radial basis function kernel; MLP (Neural Net), multilayer perceptron neural network.

**Table 3:** Classification models’ calibration performance and net benefit.

Model	Brier Score	Net Benefit Range
Logistic regression	0.377	0.10–0.50
RF	0.360	0.10–0.60
SVM-RBF	0.357	0.10–0.50
MLP (Neural Net)	0.552	0.10–0.50
XGBoost	0.482	0.10–0.50

Legend: Brier score; Net benefit range; SVM-RBF, support vector machine with radial basis function kernel; MLP (Neural Net), multilayer perceptron neural network; XGBoost; RF, Random Forest.

**Table 4:** Performance of the SVM-RBF model in the held-out test cohort.

Model	AUC	C-Index	Integrated Brier Scores
Cox PH	0.5561	0.548	0.244
Random survival forest	0.4099	0.431	0.243

Legend: AUC; C-index; Integrated Brier Score; Cox PH; random survival forest.

## Data Availability

The datasets are available from the corresponding authors upon reasonable request, due to local policies.

## Supplementary Materials

The following supporting information can be downloaded at https://www.mdpi.com/article/10.3390/jcm14228216/s1. Table S1: Missing Values per Column.

## Abbreviations

The following abbreviations are used in this manuscript:

ABC	activated B-cell
AI	artificial intelligence
AUC	Area Under the ROC Curve
CHT	chemotherapy
CI	confidence interval
CMV	cytomegalovirus
CNS	central nervous system
CNS-IPI	Central Nervous System–International Prognostic Index
CR	complete response
CT	computed tomography
DCA	decision curve analysis
DLBCL	Diffuse Large B-Cell Lymphoma
EBV	Epstein–Barr virus
ECOG	Eastern Cooperative Oncology Group (performance status)
GBM	Gradient Boosting Machine
GCB	germinal center B-cell
HBV	hepatitis B virus
HCV	hepatitis C virus
HIV	human immunodeficiency virus
IBS	Integrated Brier Score
IPI	International Prognostic Index
LDH	lactate dehydrogenase
McPM	molecular-contained prognostic model
MLP	multilayer perceptron
NHL	Non-Hodgkin Lymphoma
OS	overall survival
PET	positron emission tomography
PET-CT	positron emission tomography–computed tomography
PFS	progression-free survival
PH	proportional hazards (as in “Cox PH”)
RF	Random Forest
ROC	receiver operating characteristic
RSF	Random Survival Forest
RST	rule set table
SD	standard deviation; stable disease (context-dependent)
SEER	Surveillance, Epidemiology, and End Results (Program)
Se	sensitivity
Sp	specificity
SVM	Support vector machine
SVM-RBF	Support vector machine with radial-basis-function kernel
ULN	upper limit of normal
XGBoost	Extreme Gradient Boosting
