# Supplementary material for: Mortality Prediction in Diffuse Large B-Cell Lymphoma Using Supervised Machine Learning Models—A Retrospective Study
Source: J Clin Med. 2025 Nov 19;14(22):8216. doi: 10.3390/jcm14228216 (PMC12653685; doi:10.3390/jcm14228216)
Supplement: Supplementary file 1 [file jcm-14-08216-s001.zip › jcm-3959958-supplementary.pdf]

Table S1. Missing Values per Column.

Variable	Missing Values
Sex	0
Age_at_diagnosis	0
Comorbidities	0
Previous_neoplasm	0
Viral_hepatitis	0
HIV	0
Stage_at_diagnosis	0
B_symptoms	0
IPI	0
Extranodal_location	0
Transformation	0
Bulky_disease	0
ECOG	0
LDH	0
Bone_marrow_assessment	0
Bone_marrow_infiltration	0
CNS_infiltration_diag	0
CNS_IPI	0
CNS_IPI_risk_class	0
Gastric_involvement	0
GCB	0
ABC	0
Double_expressor	0
Ki67	**202**
Death	0
Overall_survival (OS)	0
